# Does plasticity in thermal tolerance trade off with inherent tolerance? The influence of setal tracheal gills on thermal tolerance and its plasticity in a group of European diving beetles

**DOI:** 10.1016/j.jinsphys.2017.12.005

**Published:** 2018-04

**Authors:** W.C.E.P. Verberk, P. Calosi, J.I. Spicer, S. Kehl, D.T. Bilton

**Affiliations:** a Department of Animal Ecology and Physiology, Institute for Water and Wetland Research, Radboud University, Heyendaalseweg 135, 6525 AJ Nijmegen, The Netherlands; b Département de Biologie, Chimie et Géographie, Université du Québec à Rimouski, 300 Allée des Ursulines, Rimouski, Québec G5L 3A1, Canada; c Marine Biology and Ecology Research Centre, School of Marine Science and Engineering, University of Plymouth, Davy Building, Drake Circus, Plymouth PL4 8AA, United Kingdom; d University of Applied Forest Sciences Rottenburg, Schadenweilerhof, 72108 Rottenburg a.N., Germany

**Keywords:** Acclimation, Dytiscidae, Ecophysiology, Heat tolerance, Hypoxia

## Abstract

In the face of global warming, both the absolute thermal tolerance of an ectotherm, and its ability to shift its tolerance level via acclimation, are thought to be fundamentally important. Understanding the links between tolerance and its plasticity is therefore critical to accurately predict vulnerability to warming. Previous studies in a number of ectotherm taxa suggest trade-offs in the evolution of thermal tolerance and its plasticity, something which does not, however, apply to *Deronectes* diving beetles, where these traits are instead positively correlated. Here we revisit the relationship between thermal tolerance and plasticity in these beetles, paying attention to a recently discovered morphological adaptation supporting under water respiration – setal tracheal gills. Hollow setae on the elytra interconnect with the beetle’s tracheal system, providing a gas exchange surface that allows oxygen to be extracted directly from the water. This enables individuals to stay submerged for longer than their subelytral air stores would allow. We show that hypoxia reduced heat tolerance, especially when individuals were denied access to air, forcing them to rely solely on aquatic gas exchange. Species with higher densities of these gas-exchanging setae exhibited improved cold tolerance, but reduced heat tolerance and lower plasticity of heat tolerance. Differences in setal tracheal gill density across species were also related to habitat use: species with low gill density were found mainly in intermittent, warmer rivers, where underwater gas exchange is more problematic and risks of surfacing may be smaller. Moreover, when controlling for differences in gill density we no longer found a significant relationship between heat tolerance and its plasticity, suggesting that the previously reported positive relationship between these variables may be driven by differences in gill density. Differences in environmental conditions between the preferred habitats could simultaneously select for characteristic differences in both thermal tolerance and gill density. Such simultaneous selection may have resulted in a non-causal association between cold tolerance and gill density. For heat tolerance, the correlations with gill density could reflect a causal relationship. Species relying strongly on diffusive oxygen uptake *via* setal tracheal gills may have a reduced oxygen supply capacity and may be left with fewer options for matching oxygen uptake to oxygen demand during acclimation, which could explain their reduced heat tolerance and limited plasticity. Our study helps shed light on the mechanisms that underpin thermal tolerance and plasticity in diving air-breathing ectotherms, and explores how differences in thermal tolerance across species are linked to their selected habitat, morphological adaptations and evolutionary history.

## Highlights

• Heat tolerance and its plasticity are positively correlated in *Deronectes* beetles.
• *Deronectes* beetles have setal tracheal gills which support under water respiration.
• Gill density may explain differences in thermal tolerance and habitat use.
• Habitat conditions may have simultaneously selected for cold tolerance and gills.
• Gill density may underlie the relation between heat tolerance and its plasticity.

## Graphical abstract

**Figure:**
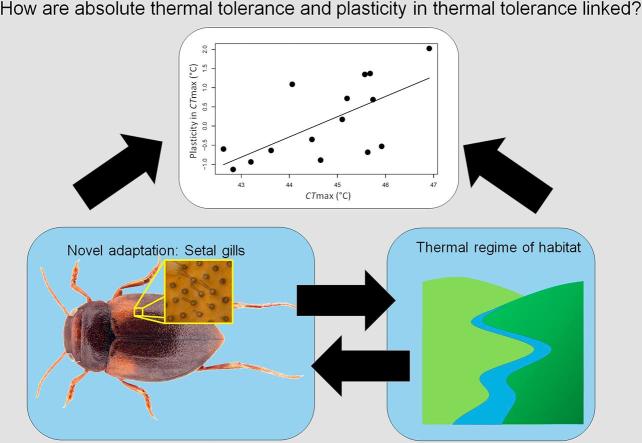

## 1 Introduction

**Fig. 1:**
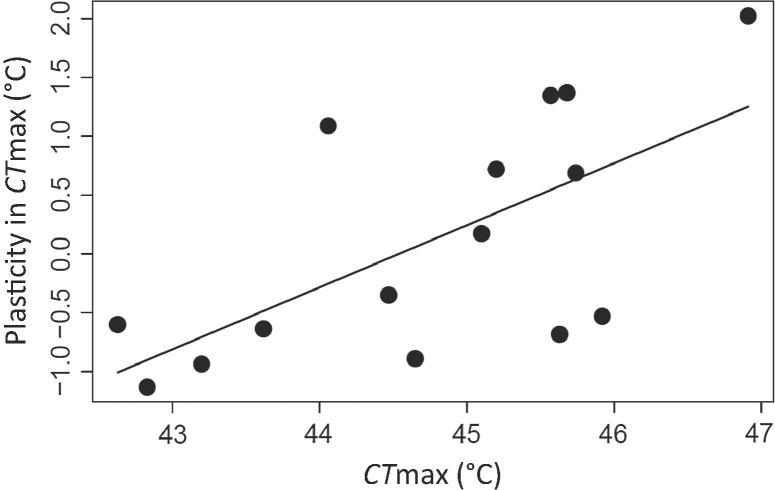
Plasticity of CTmax in relation to inherent CTmax across the 15 *Deronectes* species investigated in this study. Data for 13 of these species were previously reported by Calosi et al. (2008a). Plasticity in heat tolerance is higher for species with high inherent heat tolerance (F_1,13_ = 9.69; *P* = 0.0082).

Global warming is recognized to have profound effects on ectothermic animals. For these organisms, temperature can be considered a master control variable, as it directly affects their metabolism, growth, fecundity and survival, which in turn affects population growth rates, biodiversity, and biogeography. To respond to global warming, both the overall level of tolerance to thermal extremes (i.e., inherent thermal tolerance) and the ability to shift this in response to acclimation (i.e., plasticity of thermal tolerance) are considered fundamentally important (Stillman, 2003, Somero, 2010, Huey et al., 2012, Gunderson and Stillman, 2015). Stillman (2003) compared different species of porcelain crabs and found that species with high inherent heat tolerance exhibited reduced plasticity in heat tolerance. This led him to suggest that these two traits are connected *via* an evolutionary trade-off. A similar relationship was more recently documented for caridean shrimps, another group of crustaceans (Magozzi and Calosi, 2015). In contrast, no such trade-off was found within *Deronectes* diving beetles, where heat tolerant species actually showed greater plasticity, i.e., the opposite pattern (Calosi et al., 2008a, see Fig. 1). There may be more than one reason for this difference amongst arthropod groups. Rather than there being a direct trade-off between thermal tolerance and plasticity, both traits could have evolved in response to the thermal regime of the habitat a species occupies. Southwood (1977) proposed that the habitat provides a templet on which evolution acts to forge the characteristic traits of an organism, so that it can effectively deal with the conditions experienced. In this case, species experiencing more variable temperatures could be expected to display greater plasticity of thermal tolerance (e.g., Janzen, 1967). In a related vein, it has been suggested that the difference could be related to the direction of colonization from one habitat to another and the associated change in thermal regime (Bozinovic et al., 2011). Indeed, the ancestral habitat of the porcelain crabs was cool and stable, but for the beetles this explanation requires a consideration of the timescales, since the original habitat of dytiscids was probably lentic (relatively warm and variable), but *Deronectes* have radiated in relatively cold and stable stream habitats. Also, this explanation requires that an evolutionary trajectory away from their ancestral thermal regime is coupled with a reduction in the plasticity of thermal tolerance, irrespective of whether the trajectory is towards warmer or cooler habitats.

Alternatively, the contrasting patterns between diving beetles and crustaceans may be related to differences in respiratory capacity, as capacity limitations on oxygen uptake and delivery have been shown to be linked to thermal tolerance (Winterstein, 1905, Pörtner, 2006), especially in aquatic taxa (Woods, 1999, Verberk and Atkinson, 2013, Verberk et al., 2016a). Variation in heat tolerance has been linked to mode of respiration in aquatic insects (Verberk and Bilton, 2013, Verberk and Bilton, 2015), and to evolutionary innovations in respiration in crabs (Giomi et al., 2014). In porcelain crabs, as in other malacostracans such as caridean shrimps, gills have multiple functions, being important for osmotic and ionic regulation, acid-base balance, and ammonia excretion in addition to being a site for gas exchange (Friere et al., 2008, Henry et al., 2012). Thus it is possible that these other functional demands place constraints on the capacity for gas exchange (e.g., larger gills allow faster rates of oxygen uptake, but may also increase the need for osmoregulation). If such constraints are stronger in species that already have a high capacity for gas exchange, this could generate a negative relationship between inherent thermal tolerance and its plasticity. Adult *Deronectes* beetles, like most insects, use trachea exclusively for gas exchange. There are, therefore, arguably fewer constraints in their function in this regard. Observed differences in thermal biology between arthropod groups could simply reflect a fundamental difference in how crustaceans and insects breathe.

Here we revisit the relationship between thermal tolerance and its plasticity in *Deronectes* diving beetles (see Calosi et al., 2008a), examining the possible impact of the recently discovered setal tracheal gills (Kehl and Dettner, 2009). *Deronectes* diving beetles live in fast-flowing waters and are relatively poor swimmers (Ribera et al., 1997). Hence, surfacing to replenish air stored in their subelytral space carries the risk of being swept away by currents as well as exposing beetles to predation. Furthermore, beetles would be predicted to maximize time spent submerged where feeding and mating are carried out (Calosi et al. 2012). *Deronectes* and some associated clades have evolved a unique solution to deal with this challenge. The surface of their elytra is densely covered with setae that are tracheated and link up to channels that transverse the cuticle and connect to the longitudinal tracheal trunks embedded in the elytra. These setal tracheal gills enable beetles to extract and transport oxygen from the water directly into their tracheal system (Kehl and Dettner, 2009, Madsen, 2012). The gills allows the beetles to circumvent the diffusion barrier inherent to their thick exoskeleton and enables them to perform underwater gas exchange which is functionally similar to integumental respiration seen in many other aquatic insects (Mill, 1974). Experiments covering the elytra of *Deronectes aubei aubei* with a synthetic resin to negate oxygen uptake *via* setal tracheal gills greatly reduced their ability to extract oxygen from the water, whereas non-covered animals survived submerged for over six weeks (Kehl and Dettner, 2009).

To investigate the relationships between capacity for oxygen uptake and thermal tolerance and its plasticity, we first tested whether there was a link between oxygen-limited heat tolerance and respiratory mode by comparing individuals of *D. latus* that were forced to rely exclusively on aquatic gas exchange using tracheal respiration with individuals that could also employ aerial gas exchange using surfacing. Next, within a phylogenetically controlled framework, we (i) explored the extent to which *Deronectes* species differ in the density of setal tracheal gills as a proxy for their reliance on diffusive gas exchange, and (ii) determined whether this relates to their inherent thermal tolerance and its plasticity. Building on the habitat templet concept of Southwood (1977), we also explored whether patterns in thermal tolerance and plasticity in thermal tolerance could be related to the thermal regime of the habitat individual species occupy. Whilst all the species in this study inhabit running waters, there are differences in stream temperature and flow/permanence regimes across taxa.

## 2 Methods

### 2.1 Study species

Full data on thermal tolerance, changes in thermal tolerance following exposure to elevated temperature (thermal tolerance plasticity), density of setal tracheal gills and phylogeny were obtained for 15 *Deronectes* species (see Table S1 for an overview of the species and their morphological and physiological traits). For 13 species, thermal tolerance data have been reported elsewhere (Calosi et al., 2008a, Calosi et al., 2010). In addition, in this study we included previously unpublished data for *D. brannanii* and *D. lareynii*. Data for these two species were excluded from the previous work dealing with the relationship between thermal tolerance and geographical range size as both species are island endemics with hard dispersal barriers setting geographical range limits. Specimen collection, maintenance in the laboratory and preparation for thermal trials are described in detail elsewhere (Calosi et al., 2010). Briefly, adult *Deronectes* were collected during spring and summer 2006. By collecting species from higher latitudes later in the season we standardized as much as possible for phenological differences. All individuals collected were early post-teneral adults, minimizing any possible confounding effects due to age variation. In these beetles, adults are the longest life-history stage (≥1 year), whilst larvae are short lived (ca. 1–2 months). As a result, adult beetles typically overwinter, and/or survive periodic droughts (Nilsson and Holmen, 1995), and likely experience the greatest thermal challenges. All species were collected as close as possible to the central point of their latitudinal ranges, to avoid the possible confounding effects of local adaptation in range edge populations, and to ensure data were comparable across species (Thompson et al., 1999).

After collection individuals were transported to the laboratory where they were maintained in aquaria (vol. = 5 L, maximum 20 indiv. *per* aquarium) with aerated artificial pond water under a 12: 12 h L/D regime, and fed chironomid larvae *ad libitum*. For each species, specimens were equally divided haphazardly into two equal groups, exposed for 7 d to either 14.5 or 20.5 °C respectively before experiments were conducted. Extreme exposure temperatures were avoided and acclimation was in most cases not stressful (see Calosi et al., 2010), and indeed no mortality occurred in any species during the exposure period.

### 2.2 Thermal tolerance and its plasticity

After the exposure period, individuals from each acclimation temperature were haphazardly assigned to two equal subgroups used to determine their tolerance to heat and cold. Full methodology is described in Calosi et al., 2008a, Calosi et al., 2008b, Calosi et al., 2010). In short, thermal tolerance to cold (CTmin) and heat (CTmax) were determined using a dynamic method, by heating or cooling individuals, *via* a ramping program (±1 °C min^−1^). Ramping trials commenced at the temperature to which individuals had been acclimated. Up to 12 individuals were placed in 24 well (diam. = 12 mm, depth = 18 mm) plastic culture plates, and in turn these were placed in the water baths. Temperature within wells was measured using a digital thermometer (Omega® HH11; Omega Engineering Inc., CT, USA) with a precision fine wire thermocouple (accuracy of 0.1 °C). The wells did not contain water and hence the animals did not have to exhibit surfacing behavior for aerial gas exchange. In our analyses we employed lethal endpoints, since these showed the lowest variance amongst all end-points recorded. When animals lost responsiveness they were considered to have entered a heat or chill coma and eventually died. Plasticity in upper and lower thermal tolerance were estimated following Stillman (2003) as the absolute difference in tolerance (CTmax or CTmin) between both acclimation temperatures. A positive value for either plasticity of cold or heat tolerance indicates an improved critical temperature (higher CTmax following acclimation at the higher temperature and lower CTmin following acclimation at the lower temperature). Inherent, or overall level of tolerance against thermal extremes is given by the absolute critical temperatures and so we have two measures of inherent tolerance, one for each acclimation temperature. In our results we focused on the inherent thermal tolerance for both CTmax and CTmin displayed by animals acclimated to a common temperature of 20.5 °C, but results for both acclimation temperatures are reported in full (Table 1).

**Table 1:** Summary of OLC and PGLS analyses for *CT*max in individuals acclimated either to 20.5 °C (A), or 14.5 °C (B), *CT*min in individuals acclimated either to 20.5 °C (C), or 14.5 °C (D), plasticity in *CT*max (E) and plasticity in *CT*min (F). Gill density was measured in non-punctate regions.

Models	Coefficients	df (num, den)	Estimate	SE	p	R2
*A) CTmax in 20C acclimated animals*						
OLS: gill density	Intercept	1,13	51.22	1.05	<**0.0001**	74.9%
	Gill density	1,13	−0.00132	0.00021	<**0.0001**	
OLS: gill density + body size	Intercept	1,12	50.81	1.56	<**0.0001**	75.1%
	Gill density	1,12	−0.00131	0.00022	**0.0001**	
	Body size	1,12	0.0419	0.114	0.7186	
OLS: gill density/body size	Intercept	1,13	47.41	0.94	<**0.0001**	40.2%
	Gill density/body size	1,13	−0.00414	0.00140	**0.0112**	
PIC: gill density	Gill density	1,13	−6.321	1.202	**0.0002**	68.0%
PIC: gill density + body size	Gill density	1,12	−6.313	1.239	**0.0003**	68.6%
	Body size	1,12	0.450	0.937	0.6400	
PIC: gill density/body size	Gill density/body size	1,13	−2.593	1.052	**0.0284**	31.8%

*B) CTmax in 14.5C acclimated animals*						
OLS: gill density	Intercept	1,13	46.32	1.30	<**0.0001**	14.0%
	Gill density	1,13	−0.00038	0.00026	0.1689	
OLS: gill density + body size	Intercept	1,12	45.66	1.92	<**0.0001**	15.6%
	Gill density	1,12	−0.00036	0.00028	0.2195	
	Body size	1,12	0.0670	0.140	0.6407	
OLS: gill density/body size	Intercept	1,13	45.36	0.77	<**0.0001**	10.6%
	Gill density/body size	1,13	−0.00142	0.00115	0.2370	
PIC: gill density	Gill density	1,13	−1.132	1.394	0.4313	4.8%
PIC: gill density + body size	Gill density	1,12	−1.125	1.440	0.4501	6.2%
	Body size	1,12	0.456	1.090	0.6828	
PIC: gill density/body size	Gill density/body size	1,13	−0.701	0.835	0.4164	5.1%

*C) CTmin in 20C acclimated animals*						
OLS: gill density	Intercept	1,13	−2.29	2.48	0.3714	30.8%
	Gill density	1,13	−0.00120	0.00050	**0.0317**	
OLS: gill density + body size	Intercept	1,12	−3.99	3.63	0.2925	33.2%
	Gill density	1,12	−0.00114	0.00052	**0.0495**	
	Body size	1,12	0.1728	0.264	0.5250	
OLS: gill density/body size	Intercept	1,13	−5.55	1.54	**0.0032**	19.5%
	Gill density/body size	1,13	−0.00409	0.00230	0.0990	
PIC: gill density	Gill density	1,13	−6.425	2.386	**0.0184**	35.8%
PIC: gill density + body size	Gill density	1,12	−6.407	2.449	**0.0226**	37.5%
	Body size	1,12	1.070	1.853	0.5743	
PIC: gill density/body size	Gill density/body size	1,13	−3.021	1.577	0.0776	22.0%

*D) CTmin in 14.5C acclimated animals*						
OLS: gill density	Intercept	1,13	−3.37	2.77	0.2459	17.1%
	Gill density	1,13	−0.00091	0.00056	0.1252	
OLS: gill density + body size	Intercept	1,12	−8.47	3.58	**0.0355**	37.8%
	Gill density	1,12	−0.00072	0.00051	0.1859	
	Body size	1,12	0.5190	0.260	0.0692	
OLS: gill density/body size	Intercept	1,13	−4.70	1.50	**0.0078**	26.8%
	Gill density/body size	1,13	−0.00489	0.00224	**0.0481**	
PIC: gill density	Gill density	1,13	−3.734	2.858	0.2141	11.6%
PIC: gill density + body size	Gill density	1,12	−3.671	2.594	0.1825	32.8%
	Body size	1,12	3.818	1.962	0.0755	
PIC: gill density/body size	Gill density/body size	1,13	−3.764	1.495	**0.0257**	32.8%

*E) delta CTmax*						
OLS: gill density	Intercept	1,13	4.69	1.11	**0.0010**	57.4%
	Gill density	1,13	−0.00093	0.00022	**0.0011**	
OLS: gill density + body size	Intercept	1,12	5.18	1.64	**0.0082**	58.0%
	Gill density	1,12	−0.00095	0.00024	**0.0016**	
	Body size	1,12	−0.0505	0.119	0.6794	
OLS: gill density/body size	Intercept	1,13	1.70	0.86	0.0704	22.1%
	Gill density/body size	1,13	−0.00248	0.00129	0.0771	
PIC: gill density	Gill density	1,13	−5.463	1.089	**0.0002**	65.9%
PIC: gill density + body size	Gill density	1,12	−5.471	1.121	**0.0004**	66.7%
	Body size	1,12	−0.436	0.848	0.6163	
PIC: gill density/body size	Gill density/body size	1,13	−1.723	1.012	0.1123	18.2%

*F) delta CTmin*						
OLS: gill density	Intercept	1,13	1.07	1.65	0.5268	5.4%
	Gill density	1,13	−0.00029	0.00033	0.4038	
OLS: gill density + body size	Intercept	1,12	4.48	2.03	**0.0481**	35.0%
	Gill density	1,12	−0.00042	0.00029	0.1788	
	Body size	1,12	−0.3462	0.148	**0.0375**	
OLS: gill density/body size	Intercept	1,13	−0.85	0.96	0.3957	2.3%
	Gill density/body size	1,13	0.00080	0.00144	0.5876	
PIC: gill density	Gill density	1,13	−2.691	1.801	0.1591	14.7%
PIC: gill density + body size	Gill density	1,12	−2.736	1.554	0.1037	41.4%
	Body size	1,12	−2.748	1.176	**0.0375**	
PIC: gill density/body size	Gill density/body size	1,13	0.743	1.151	0.5297	3.1%

Significant *P* values are in boldface.

### 2.3 Effect of oxygen availability on heat tolerance in *D. latus*

We assessed the impact of mode of respiration on heat tolerance under different oxygen conditions in one of the 15 species: *D. latus*, the most tolerant species in our comparison, using previously described methods (Verberk and Calosi, 2012, Verberk and Bilton, 2015). Briefly, individuals were placed in flow-through chambers, whose water supply could be heated. For one group of animals, we used chambers where the animals were completely submerged and had no access to air, while for a second group of animals chambers were used with a small head space holding a layer of air, meaning that these animals could obtain oxygen either from the air compartment by surfacing or from the water with oxygen diffusing directly into their tracheal system *via* the setae or oxygen diffusing into their subelytral air reservoir *via* their physical gill. Individuals were left to settle for 1 h at the equilibration temperature of 10 °C, after which the temperature was ramped up at 0.25 °C min^−1^. The CTmax was defined as the point at which animals lost coordinated swimming, hence losing their ability to escape from the conditions that will lead to their death (Lutterschmidt and Hutchison, 1997). The heating rate, endpoint and starting temperature all therefore differed from the methodology described above, meaning that the critical thermal temperatures from both methods cannot be compared directly. CTmax was assessed under normoxia, hypoxia and hyperoxia conditions (5, 20, 60 kPa O_2_ respectively) and adults were assessed with and without access to air. Oxygen tension of both the water and the air in the headspace was altered to produce hypoxia and hyperoxia, as described by Verberk and Bilton (2015).

### 2.4 Setal tracheal gill enumeration

**Fig. 2:**
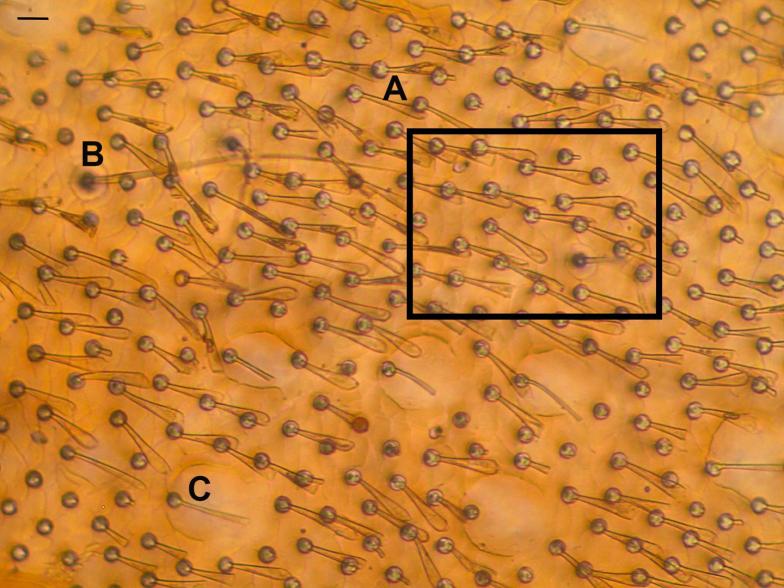
Elytral punctation and setation in *Deronectes aubei aubei*. Setal tracheal gills are spoon-shaped and flattened (A), corresponding to a form of sensillum trichoideum type 2 of Wolfe and Zimmermann (1984). Also visible are scattered examples of sensillum trichoideum type 1 (B) and rod-like setae associated with large punctures (C – also a form of sensillum trichoideum type 2). Only setae of type (A) were enumerated, the rectangle highlighting an area without punctures (see text). Scale bar = 10 µm.

Species of the genus *Deronectes* possess different types of setae. The setae for which a respiratory function has been demonstrated are spoon-shaped, with an enlarged base, situated in simple punctures. In addition, beetles possess long sensory setae in punctures encircled by concentric ridges, and rod-like setae associated with deep punctures or craters (see Fig. 2). Only the spoon-like, setal tracheal gills, which were by far the most dominant type on *Deronectes* elytra, were enumerated.

Density of setal tracheal gills was determined from digital images of the elytra, using light microscopy. With the use of image acquisition software (Olympus software package “Cell^A”), the number of setae were counted in four regions of the elytra; the posterior section of the elytra (at 100x magnification, on average 0.074 mm^2^), the middle section (at 40× magnification, ca. 0.155 mm^2^) the anterior section (at 200× magnification, ca. 0.040 mm^2^), and lastly setae were enumerated in an anterior section without the deep punctation that is associated with the rod-like setae (at 200× magnification, ca. 0.0050 mm^2^). In each section, the largest relatively flat area was chosen to count setae as this ensured they all appeared in focus. The size of this area was automatically calculated by the Olympus cell program. Setal density is expressed as the number of setae *per* mm^2^. More than 100,000 setae were counted on a total of 74 individuals, five for each species, with the exception of *D. angusi* (n = 3) and *D. moestus* (n = 6).

### 2.5 Data analysis

In order to investigate the effect of oxygen tension on the CTmax of *D. latus*, we used linear models with ‘oxygen conditions’ (hypoxia, normoxia or hyperoxia) and ‘access to air’ (access or no access to air) as fixed factors. We also included the interaction between these two terms to test whether effects of oxygen on CTmax differed when individuals exposed to different oxygen levels had access to air or not. Data from these trials showed small deviations from normality (visually assessed from Q-Q plots) and homogeneity of variances (formally tested using Levene’s test), which were due to large variability in CTmax observed under hypoxia in the treatment without access to air. A conservative analysis, which excluded the three lowest values to meet test assumptions, yielded qualitatively similar results, flagging the same contrasts as being significant. We therefore deemed the analysis robust to these small deviations and present the complete results.

Differences in setal tracheal gill density across species were analyzed using a linear model with ‘species’ as a fixed factor, followed by Tukey post hoc tests. Preliminary analysis showed that the three measures of seta on the anterior, middle and posterior region were highly correlated across the 15 species (R^2^ > 0.923, t_1,13_ > 12.97; *P* < 0.0001) and also across all 74 individuals, accounting for species differences in a mixed effect model (R^2^ > 0.80; t_1,72_ > 11.51; *P* < 0.0001). Therefore these three measures of gill density were averaged to produce a composite measure (hereafter referred to as gill density in punctated sections). The fourth measure of gill density in sections without punctation was found to be correlated much less strongly to this composite measure in a mixed effect model (R^2^ > 0.13; t_1,72_ > 3.707; *P* = 0.00021) and was therefore analyzed separately (hereafter referred to as gill density). Preliminary analyses also showed that variation in setal density of individuals was mainly due to interspecific differences rather than body size and sex. When included in a mixed effect model to explain intraspecific differences in gill density, elytra length (as a measure of body size) was not significant (t_1,72_ = 0.145; *P* = 0.885). Similar results were obtained for the composite measure of gill density in punctated sections (t_1,72_ = −0.080; *P* = 0.936). Furthermore, across the 15 species, body size was not significantly related to gill density (t_1,13_ = −0. 700; *P* = 0.496) nor gill density in punctated sections (t_1,13_ = −0. 899; *P* = 0.385). Similar non-significant results were found when including sex in a mixed effect model on individuals (t_1,72_ > −1.505; *P* > 0.13), indicating that gill density did not differ between males and females.

Relationships between a species mean gill density, mean thermal tolerance (CTmax and CTmin) and plasticity in thermal tolerance were analyzed using linear regressions across the 15 *Deronectes* species. To test whether the same outcome was obtained within a phylogenetically controlled framework, we also analyzed the relationships between gill density, CTmax, and plasticity in CTmax using phylogenetic independent contrasts, in the R-package {ape} (Paradis et al. 2004). Independent contrasts were derived from DNA based phylogenies (García-Vázquez et al., 2016). Preliminary analyses showed that none of the thermal tolerance traits exhibited a strong phylogenetic signal (K < 0.37; see Blomberg et al., 2003). The same was true for both measures of gill density (K < 0.39). We therefore opted to rescale the tree using a lambda of 0.5, representing the intermediate between a Brownian evolution model and a star phylogeny. Diagnostic tests were performed using the function {caic.diagnostics} from the R-package {caper} (Orme et al., 2011). These diagnostics showed that the estimated nodal values correlated with the magnitude of the estimated contrasts, a problem that was solved by log-transformation of the data on gill density in the phylogenetic independent contrast analyses.

Variation in habitats used by *Deronectes* species was condensed into two categories: permanent streams, often at high altitudes, which tend to be cooler, often faster flowing and thermally more constant (constant streams) and streams which may be intermittent, have lower flow and exhibit higher and more widely fluctuating temperatures (fluctuating streams). Species primarily inhabiting the permanent streams are *D. angusi, D. aubei aubei, D. bicostatus, D. depressicollis, D. lareynii, D. platynotus platynotus, D. semirufus and D. wewalkai*. Species primarily inhabiting the warmer, mostly intermittent, streams are *D. algibensis, D. brannanii, D. latus, D. fairmairei, D. hispanicus, D. moestus and D. opatrinus*. Differences in gill density between species occupying the two habitat types were assessed using a *t*-test.

## 3 Results

### 3.1 Heat tolerance of *Deronectes latus* in relation to respiratory mode

**Fig. 3:**
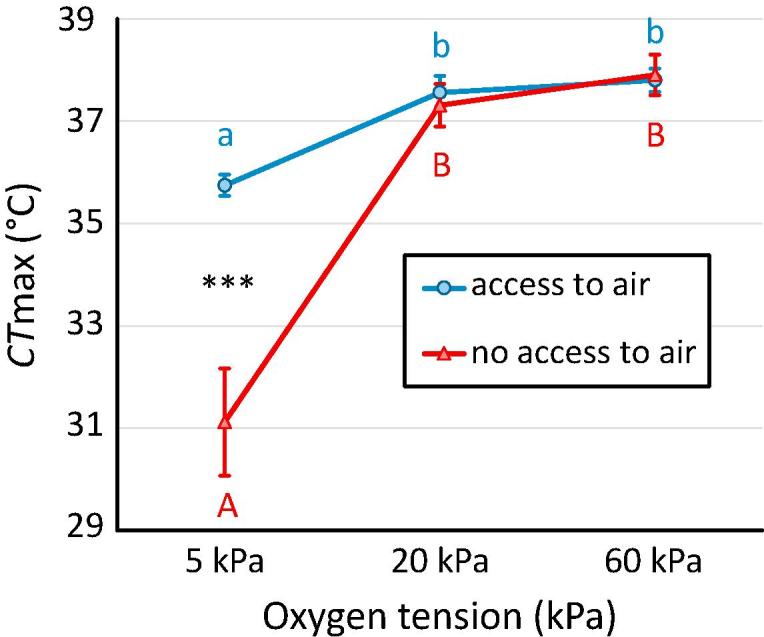
Mean heat tolerance of *Deronectes latus* at different oxygen tensions. Treatment differences with (circles, in blue) and without (triangles, in red) access to air are shown separately. Letters indicate differences between oxygen levels within treatment and asterisks indicate differences between treatments within oxygen levels. Error bars indicate SEs (n = 9 in all cases except for the normoxia and hyperoxia treatments without access to air where n = 10).

In *Deronectes latus*, CTmax was reduced by 1.8 °C in hypoxia (5 kPa), compared to normoxia (20 kPa). This reduction increased to 6.2 °C for individuals denied access to air (Fig. 3).

### 3.2 Density of setal tracheal gills

**Fig. 4:**
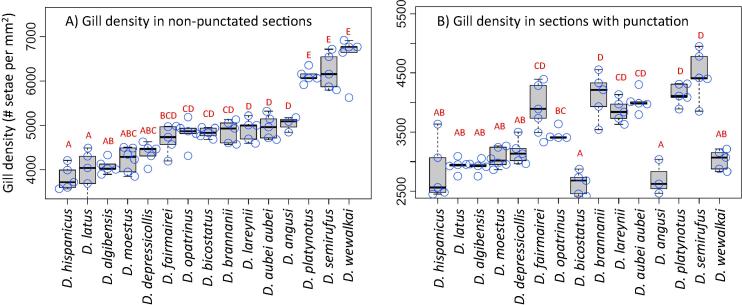
Setal tracheal gill densities in *Deronectes* species. A) Density measured in a section without punctation, and B) Density in sections with punctation, averaged across anterior, middle and posterior sections of the beetle’s elytra (see methods). Different letters indicate significant differences between species (*P* < 0.05). Individuals are indicated by blue circles to illustrate the spread and distribution of data.

There were clear differences between species in mean gill density (Fig. 4A). These differences were found to be significant (GLM: Species F_15,74_ = 33.74; *P* < 0.0001), with average densities (# seta per mm^2^) varying from 3444 in *D. hispanicus* to 6680 in *D. wewalkai*. Differences across species in gill density in punctated sections were smaller (Fig. 4B; F_15,74_ = 23,18; *P* < 0.0001) and this measure of gill density had a higher coefficient of variation (7.2% vs 5.9%). Differences between density in punctated and non-punctated regions were greatest in *D. bicostatus*, *D. angusi* and *D. wewalkai*. As variation in punctation is likely related to differences in flow sensory ability, we focus subsequent analyses on non-punctated sections (see Table 1), which was not confounded by the degree of punctation and report results on setal density in punctated regions in Table S2.

### 3.3 Thermal tolerance in relation to setal tracheal gill density

**Fig. 5:**
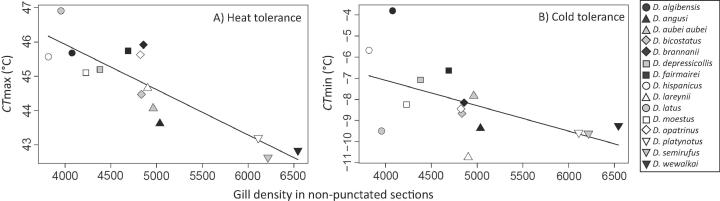
Relationship between gill density and mean CTmax (A) and CTmin (B) in 15 *Deronectes* species, acclimated to 20.5 °C. A) Trendlines indicate significant relationships (*P* < .05).

In beetles acclimated to 20.5 °C, species with high gill density had significantly reduced CTmax (F_1,13_ = 38.72; *P* < 0.0001; R^2^ = 0.75; Fig. 5A), and improved (i.e., lower) CTmin (F_1,13_ = 5.79; *P* = 0.0318; R^2^ = 0.308; Fig. 5B). There was no significant relationship between gill density and the difference between CTmin and CTmax (*P* = 0.505), indicating that the thermal window shifted with gill density, rather than widening or narrowing. The relationships between gill density and thermal tolerance were upheld when phylogenetic non-independence was accounted for (Table 1). No significant relationship was detected between thermal tolerance and gill density for individuals acclimated to 14.5 °C, neither for heat tolerance (*P* = 0.169) nor cold tolerance (*P* = 0.125). Also, no significant relationship was detected between thermal tolerance and the composite measure of gill density in punctated regions (Table S2; P > 0.31). Furthermore, analyses accounting for differences in body size by including body size as a covariate showed that size did not have a significant effect (*P* > 0.063). When gill density was expressed on a size-specific basis, we found a significant relationship for CTmax only (*P* = 0.0112), where species with a relatively high gill density had reduced CTmax.

### 3.4 Plasticity in thermal tolerance in relation to gill density

**Fig. 6:**
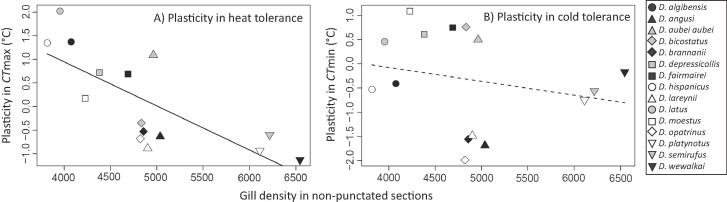
Relationship between gill density and mean plasticity of thermal tolerance for CTmax (A) and CTmin (B) in 15 *Deronectes* species. Trendlines indicate significant relationships (*P* < 0.05).

Plasticity in thermal tolerance (i.e., the change in critical temperatures with acclimation) was related to gill density. Beetles with a higher gill density showed greater plasticity in CTmax (F_1,13_ = 17.48; *P* = 0.0011; R^2^ = 0.574; Fig. 6A), but not CTmin (*P* = 0.412; Fig. 6B). As noted in the introduction, inherent heat tolerance and plasticity in heat tolerance were also correlated across these *Deronectes* species (Fig. 1). Therefore, we used partial regressions to factor out any confounding influences. This analysis still revealed an effect of gill density on plasticity in CTmax, when controlling for CTmax (*P* = 0.046). In contrast, we found no significant relationship between CTmax and plasticity in CTmax after controlling for the effect of gill density (*P* = 0.98). When applying phylogenetic independent contrasts, an even stronger relationship between gill density and plasticity in thermal tolerance was found for CTmax (*P* = 0.00023; R^2^ = 0.659), but the relationship remained non-significant for CTmin (*P* = 0.220) (Fig. S1). Plasticity in thermal tolerance was found to be unrelated to the composite measure of gill density in punctated regions (Table S2; *P* > 0.282). Also, accounting for differences across species in body size did not reveal an effect on plasticity in CTmax (*P* = 0.679), but larger species did show lower plasticity in CTmin (*P* = 0.0480). When gill density was expressed on a size specific basis, we found no significant effects on plasticity in CTmax or CTmin (*P* > 0.0771).

### 3.5 Habitat use

**Fig. 7:**
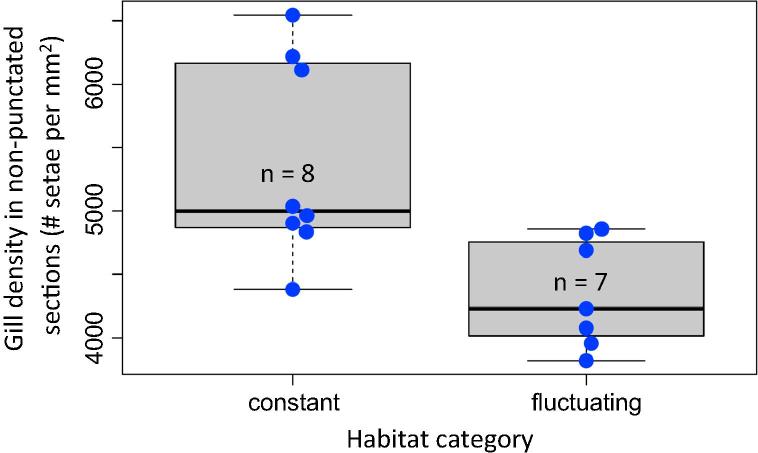
Difference in mean setal tracheal gill density between *Deronectes* species inhabiting cool, thermally constant streams and warmer, thermally variable streams. Species are indicated by blue circles to illustrate the spread and distribution of the data.

Species of the two habitat categories differed in their gill density (Fig. 7; t_1,13_ = −3.034; *P* = 0.0096); taxa associated with thermally constant streams having higher gill densities than those from thermally fluctuating habitats (see methods for habitat categorization).

## 4 Discussion

Southwood (1977) highlighted habitat as a driving force for evolutionary adaptations, acting as a templet on which evolution acts to forge characteristic traits enabling an organism to survive in its environment. Here we show how differences in gill density are associated with ecological differences in habitat use in *Deronectes* diving beetles, and that these are correlated to physiological differences in thermal tolerance and plasticity. Correlation does not, of course, equate to causation, and the associations demonstrated in this study could arise in a number of ways. Differences in environmental conditions could simultaneously select for differences in thermal tolerance and gill density, resulting in a non-causal association between these traits. Alternatively, gill density could shape thermal tolerance directly, reinforcing any correlation between these two characteristics.

Differences in thermal tolerance between *Deronectes* species have been related to aspects of their geographic range (extent, northern and southern limits – see Calosi et al., 2010), suggesting that the thermal regime of the habitat is indeed related to thermal tolerance. The CTmax and CTmin observed in short-term ramping experiments are unlikely to match the temperatures that the beetles would normally experience in the field, but are best viewed as proxies for the temperatures that species can tolerate *in situ*. Indeed, a recent study on mayflies showed interactive effects between warming and hypoxia for both lethal temperatures in short-term laboratory ramping experiments and sublethal temperatures experienced in the field, suggesting a commonality of the physiological mechanisms involved in both lethal and sublethal thresholds (Verberk et al., 2016b).

Habitat conditions may also directly select for differences in gill density. The evolution of high densities of setal tracheal gills within the *Deronectes* group suggests that staying submerged is an adaptive strategy in these largely lotic aquatic insects. Presence/absence and variation in gill density may capture a gradient from beetles relying completely on aerial gas exchange *via* surfacing to beetles relying on diffusive oxygen uptake, enabled by dense setal tracheal gills which allow beetles to remain submerged for longer (Kehl and Dettner, 2009). Higher gill densities, enabling more oxygen uptake, could be argued to be more important in warmer habitats where beetles require more oxygen, yet we found high densities to be associated with cold, stable, permanent flowing waters (Fig. 7). Being more reliant on diffusive oxygen uptake carries the disadvantage of reduced capacity to regulate oxygen uptake, making beetles more prone to oxygen limitation (Verberk and Bilton, 2013, Verberk and Atkinson, 2013). Both fast flow leading to thinner boundary layers and cool water reduce the risk of asphyxiation. Warm, intermittent streams are often reduced to isolated pools of standing water in the summer, which can warm up dramatically. Under these conditions, aerial gas exchange by surfacing represents a more convenient respiratory strategy when compared to under water gas exchange. Other aquatic insects that rely on diffusive oxygen uptake *via* a plastron likewise depend on cold, flowing water (Jones et al., 2018) and are more prone to oxygen limitation (Verberk and Bilton, 2015).

Given that habitat conditions likely influence both thermal tolerance and gill density, the key question is whether these two characteristics are mechanistically linked. This could be different for cold and heat tolerance, as the underlying mechanisms may differ with mechanisms other than oxygen limitation being more important in cold tolerance (Hoffmann et al., 2002, Stevens et al., 2010, Verberk et al., 2016a). A relationship between CTmin and respiratory structures is therefore less likely and indeed the observed relationships with gill density are weaker for CTmin than for CTmax (Table 1). Thus, we believe that differences in cold tolerance may predominantly reflect selection pressures originating from the different habitat conditions and that the correlation between cold tolerance and gill density is non-causal. The concordant differences between, on the one hand, gill density and on the other heat tolerance and plasticity for heat tolerance, could also reflect selection pressures originating from the different habitat conditions, similar to the situation for cold tolerance. Alternatively, thermal tolerance traits may be directly linked to the reliance of species on diffusive gas exchange. We found that hypoxia reduced heat tolerance in *Deronectes latus*, especially when individuals were denied access to air, forcing them to solely rely on aquatic gas exchange. This indicates that aerial gas exchange by surfacing is important for *D. latus* when faced with warmer waters. In aquatic hemipterans we have similarly shown that oxygen limitation of thermal tolerance can be induced in a bimodal breather by negating aerial respiration (Verberk and Bilton 2015).

Our observations on *D. latus* point to a role of oxygen and mode of respiration in setting CTmax, but cannot explain the observed patterns in thermal tolerance across all species investigated, since thermal tolerance trials were conducted under aerial, normoxic conditions. It is possible that species which are more reliant on underwater gas exchange have lower tracheal conductance, and a reduced capacity for aerial breathing, but this has not yet been verified experimentally. The strong negative correlation between plasticity of heat tolerance and gill density is suggestive of a direct relationship, although it is not immediately obvious how plasticity of heat tolerance and gill density would be linked mechanistically. It is possible that high reliance on diffusive oxygen uptake *via* setal tracheal gills provides fewer options for matching oxygen uptake to oxygen demand, which could in turn limit plasticity for heat tolerance. One way to increase diffusive oxygen uptake is to maintain steeper gradients in *p*O_2_ but obviously there are limits to how far internal *p*O_2_ can be lowered in practice. Lane et al. (2017) show that such limits can explain maximum body sizes in pycnogonids, which also rely on gas exchange across their cuticle. As individual species may differ in the thermal windows over which they can effectively acclimate (Calosi et al., 2010), it is also possible that using the same acclimation temperatures across all species may have underestimated plasticity of heat tolerance in species with high gill density, which typically occupy cooler habitats.

Strong relationships were found for gill density in non-punctated sections of the elytra, but not in punctated regions (Table S2). Punctures and associated setae may have a sensory function, meaning that their densities and distribution are driven by selection pressures unrelated to gas exchange. Punctures take up surface area that cannot be covered by setal tracheal gills and the density of gills in punctated sections may be driven largely by non-respiratory factors. Gill density in non-punctated sections of the elytra may better reflect selection to increase capacity for underwater gas exchange, and could be accompanied by other physiological changes to further increase supply capacity (e.g., a lower internal *p*O_2_). Since the coldest habitats are also characterised by faster flow and more stable discharge, it is difficult to disentangle the selection pressures on gill density and heat tolerance. Seebacher (2015) reported greater plasticity in freshwater species from more thermally variable, warmer habitats, which would support the explanation that variation in heat tolerance across species is driven by the thermal regime of their preferred habitat (see also Gaston et al., 2009, Bozinovic et al., 2011). What is clear though is that beetles with high gill density prefer cold, fast flowing waters. Here, underwater gas exchange by diffusion can be sufficient to sustain the low metabolic demands and enable prolonged submergence. The thermal regime of the preferred habitat of these beetles matches their relatively low heat tolerance and plasticity.

Our study contributes to our overall understanding of thermal tolerance and plasticity in ectotherms by linking such differences across species to their morphological adaptations, whilst controlling for their evolutionary history. Our work on *Deronectes* shows that heat tolerance and plasticity need not be negatively correlated, suggesting that the postulated trade-off does not exist or can at least be circumvented. The previously reported positive relationship between inherent heat tolerance and plasticity in *Deronecte*s beetles may be driven by differences in gill density, as no relationship between inherent heat tolerance and plasticity remained after accounting for differences in these structures. This suggests that the positive relationship observed in *Deronectes* may be an exception (Stillman, 2003, Magozzi and Calosi, 2015). As a prediction, we would not expect a positive relationship between inherent heat tolerance and plasticity in beetles that do not possess setal tracheal gills and instead use aerial respiration. Indeed using published and unpublished data for 13 species of the dytiscid tribe Agabini (see Calosi et al., 2008a), no relationship was detected between plasticity and inherent heat tolerance either in individuals acclimated at 20.5 °C (β = 0.451; F_1,11_ = 4.30; *P* = 0.062; R^2^ = 0.22) or 14.5 °C (β = −0.332; F_1,11_ = 1.57; *P* = 0.236; R^2^ = 0.05).
