# Supplementary material for: Does plasticity in thermal tolerance trade off with inherent tolerance? The influence of setal tracheal gills on thermal tolerance and its plasticity in a group of European diving beetles
Source: J Insect Physiol. 2018 Apr;106:163–71. doi: 10.1016/j.jinsphys.2017.12.005 (PMC5968350; doi:10.1016/j.jinsphys.2017.12.005)
Supplement: Supplementary data 1 — Figure S1, Tables S1 and S2 [file mmc1.docx]

Figure S1. Relationship between setal tracheal gill density and plasticity in either heat tolerance (A) or cold tolerance (B) evaluated by phylogenetic independent (PI) contrasts. Note that for the plasticity in heat tolerance, the relationship based on PI contrasts is stronger than that based on OLS (see Fig. 6A).

Table S1. Overview of the body size, collection locality, gill density and thermal tolerance for the 15 species of *Deronectes* diving beetles investigated in this study.

| Species name | Authority | Body size (+SE) |
| --- | --- | --- |
| *D. aubei aubei* | (Mulsant, 1843) | 6.34 (+0.12) |
| *D. semirufus* | (Germar, 1845) | 8.21 (+0.17) |
| *D. platynotus* | (Germar, 1834) | 5.37 (+0.2) |
| *D. lareynii* | (Fairmaire, 1858) | 9.177 (+0.19) |
| *D. fairmairei* | (Leprieur, 1876) | 8.91 (+0.1) |
| *D. moestus* | (Fairmaire, 1858) | 6.01 (+0.19) |
| *D. brannanii* | (Schauffus, 1869) | 6.838 (+0.13) |
| *D. bicostatus* | (Schaum, 1864) | 6.82 (+0.12) |
| *D. wewalkai* | Fery & Fresneda, 1988 | 8.54 (+0.11) |
| *D. algibensis* | Fery & Fresneda, 1988 | 9.89 (+0.22) |
| *D. depressicollis* | (Rosenhauer, 1856) | 6.28 (+0.14) |
| *D. opatrinus* | (Germar, 1824) | 10.54 (+0.28) |
| *D. hispanicus* | (Rosenhauer, 1856) | 9.88 (+0.15) |
| *D. angusi* | Fery & Brancucci, 1990 | 9.14 (+0.21) |
| *D. latus* | (Stephens, 1829) | 7.96 (+0.13) |

Table S1. continued

| Species name | Habitat type | Gill density (+SE) | Gill density in punctated region (+SE) | n (gill density) |
| --- | --- | --- | --- | --- |
| *D. aubei aubei* | 1. cold, fast flowing, permanent streams with more constant temperatures | 4963.2 (+122.6) | 4013.6 (+82) | 5 |
| *D. semirufus* | 1. streams with pools of more constant temperatures and connected by groundwater | 6217.4 (+181.2) | 4478.6 (+189.1) | 5 |
| *D. platynotus* | 1. permanent streams with more constant temperatures | 6111.6 (+71.9) | 4142 (+79.5) | 5 |
| *D. lareynii* | 1. cold, fast flowing, permanent streams with more constant temperatures | 4902.2 (+113.9) | 3860.9 (+88.7) | 5 |
| *D. fairmairei* | 2. warm, intermittent, lowland streams with more fluctuating temperatures | 4689.4 (+145.9) | 3959.5 (+168.7) | 5 |
| *D. moestus* | 2. warm, intermittent, lowland streams with more fluctuating temperatures | 4228.2 (+112.3) | 3058.1 (+66.5) | 6 |
| *D. brannanii* | 2. warm, intermittent, lowland streams with more fluctuating temperatures | 4857.2 (+115.2) | 4113.9 (+175.1) | 5 |
| *D. bicostatus* | 1. cold, fast flowing, permanent streams with more constant temperatures | 4834.2 (+51.7) | 2631.9 (+86.2) | 5 |
| *D. wewalkai* | 1. cold, fast flowing, permanent streams with more constant temperatures | 6544.9 (+234.1) | 3028.5 (+74.7) | 5 |
| *D. algibensis* | 2. warm, intermittent, lowland streams with more fluctuating temperatures | 4076.2 (+73.4) | 2921.4 (+48.8) | 5 |
| *D. depressicollis* | 1. cold, fast flowing, permanent streams with more constant temperatures | 4381.4 (+104) | 3167.2 (+94.5) | 5 |
| *D. opatrinus* | 2. warm, intermittent, lowland streams with more fluctuating temperatures | 4823.1 (+142.4) | 3439.7 (+51.9) | 5 |
| *D. hispanicus* | 2. warm, intermittent, lowland streams with more fluctuating temperatures | 3818.1 (+122.5) | 2838.9 (+228.1) | 5 |
| *D. angusi* | 1. cold, permanent streams with more constant temperatures | 5036.4 (+99.9) | 2707.8 (+170.7) | 3 |
| *D. latus* | 2. warm, permanent, lowland streams | 3955.5 (+220.4) | 2933.5 (+53.6) | 5 |

Table S1. continued

| Species name | mean CTmax (+ SE) at 14.5 °C | mean CTmax (+ SE) at 20.5 °C | Plasticity in Ctmax | mean CTmin (+ SE) at 14.5 **°**C | mean CTmin (+ SE) at 20.5 °C | Plasticity in Ctmin | n (thermal tolerance) |
| --- | --- | --- | --- | --- | --- | --- | --- |
| *D. aubei aubei* | 42.97 (+0.55) | 44.06 (+0.27) | 1.0886 | -8.34 (+0.54) | -7.84 (+0.45) | 0.5014 | 28 |
| *D. semirufus* | 43.23 (+0.69) | 42.63 (+0.2) | -0.6 | -9.06 (+0.37) | -9.62 (+0.4) | -0.56 | 60 |
| *D. platynotus* | 44.14 (+0.78) | 43.2 (+0.32) | -0.9364 | -8.83 (+0.35) | -9.58 (+0.42) | -0.7497 | 47 |
| *D. lareynii* | 43.76 (+0.43) | 44.65 (+0.25) | -0.89 | -9.26 (+0.31) | -10.75 (+0.53) | -1.49 | 48 |
| *D. fairmairei* | 45.06 (+0.16) | 45.74 (+0.21) | 0.6888 | -7.39 (+0.61) | -6.64 (+0.29) | 0.7449 | 36 |
| *D. moestus* | 44.93 (+0.21) | 45.1 (+0.3) | 0.1727 | -9.34 (+0.44) | -8.25 (+0.52) | 1.0867 | 48 |
| *D. brannanii* | 45.39 (+0.16) | 45.92 (+0.15) | -0.53 | -6.60 (+0.55) | -8.16 (+0.51) | -1.56 | 73 |
| *D. bicostatus* | 44.82 (+0.34) | 44.47 (+0.27) | -0.35 | -9.43 (+0.72) | -8.67 (+0.66) | 0.7623 | 53 |
| *D. wewalkai* | 43.96 (+1.01) | 42.83 (+0.23) | -1.132 | -9.08 (+0.33) | -9.25 (+0.42) | -0.1697 | 68 |
| *D. algibensis* | 44.3 (+0.36) | 45.68 (+0.27) | 1.3695 | -3.4 (+0.8) | -3.81 (+0.47) | -0.414 | 52 |
| *D. depressicollis* | 44.48 (+0.17) | 45.2 (+0.26) | 0.72 | -7.69 (+0.36) | -7.08 (+0.52) | 0.6057 | 57 |
| *D. opatrinus* | 46.31 (+0.24) | 45.63 (+0.23) | -0.6835 | -6.46 (+1.08) | -8.45 (+0.35) | -1.9889 | 54 |
| *D. hispanicus* | 44.22 (+0.14) | 45.57 (+0.09) | 1.3465 | -5.15 (+0.34) | -5.68 (+0.29) | -0.5288 | 92 |
| *D. angusi* | 44.26 (+0.93) | 43.62 (+0.24) | -0.6371 | -7.68 (+0.82) | -9.37 (+0.52) | -1.6837 | 26 |
| *D. latus* | 44.88 (+0.18) | 46.91 (+0.28) | 2.0226 | -9.96 (+0.33) | -9.5 (+0.43) | 0.4591 | 75 |

Table S2. Summary of OLC and PGLS analyses for *CT*max in individuals acclimated either to 20.5 °C (A), or 14.5 °C (B), *CT*min in individuals acclimated either to 20.5 °C (C), or 14.5 °C (D), plasticity in *CT*max (E) and plasticity in *CT*min (F). Gill density was measured in punctate regions.

| Models | Coefficients | df (num, den) | estimate | SE | p | R2 |
| --- | --- | --- | --- | --- | --- | --- |
| **A) CTmax in 20 C acclimated animals** | |  |  |  |  |  |
| OLS: gill density | intercept | 1,13 | 46.64 | 1.90 | **0.0000** | 7.3% |
|  | gill density | 1,13 | -0.00055 | 0.00055 | 0.3307 |  |
| OLS: gill density + body size | intercept | 1,12 | 45.37 | 2.96 | **0.0000** | 9.7% |
|  | gill density | 1,12 | -0.00047 | 0.00058 | 0.4300 |  |
|  | body size | 1,12 | 0.1250 | 0.219 | 0.5788 |  |
| OLS: gill density/body size | intercept | 1,13 | 46.00 | 1.09 | **0.0000** | 10.0% |
|  | gill density/body size | 1,13 | -0.00278 | 0.00231 | 0.2505 |  |
| PIC: gill density | gill density | 1,13 | -1.521 | 2.146 | 0.4909 | 3.7% |
| PIC: gill density + body size | gill density | 1,12 | -1.491 | 2.229 | 0.5162 | 4.3% |
|  | body size | 1,12 | 0.456 | 1.639 | 0.7854 |  |
| PIC: gill density/body size | gill density/body size | 1,13 | -0.826 | 1.245 | 0.5186 | 3.3% |
| **B) CTmax in 14.5 C acclimated animals** | |  |  |  |  |  |
| OLS: gill density | intercept | 1,13 | 45.63 | 1.28 | **0.0000** | 6.4% |
|  | gill density | 1,13 | -0.00035 | 0.00037 | 0.3620 |  |
| OLS: gill density + body size | intercept | 1,12 | 44.88 | 2.00 | **0.0000** | 8.4% |
|  | gill density | 1,12 | -0.00030 | 0.00039 | 0.4570 |  |
|  | body size | 1,12 | 0.0742 | 0.148 | 0.6245 |  |
| OLS: gill density/body size | intercept | 1,13 | 45.16 | 0.74 | **0.0000** | 7.2% |
|  | gill density/body size | 1,13 | -0.00158 | 0.00157 | 0.3338 |  |
| PIC: gill density | gill density | 1,13 | -0.652 | 1.459 | 0.6625 | 1.5% |
| PIC: gill density + body size | gill density | 1,12 | -0.622 | 1.510 | 0.6877 | 2.8% |
|  | body size | 1,12 | 0.445 | 1.110 | 0.6959 |  |
| PIC: gill density/body size | gill density/body size | 1,13 | -0.508 | 0.839 | 0.5555 | 2.7% |
| **C) CTmin in 20 C acclimated animals** | |  |  |  |  |  |
| OLS: gill density | intercept | 1,13 | -5.44 | 2.68 | 0.0638 | 7.6% |
|  | gill density | 1,13 | -0.00080 | 0.00077 | 0.3194 |  |
| OLS: gill density + body size | intercept | 1,12 | -7.70 | 4.15 | 0.0885 | 11.5% |
|  | gill density | 1,12 | -0.00066 | 0.00081 | 0.4332 |  |
|  | body size | 1,12 | 0.2224 | 0.307 | 0.4830 |  |
| OLS: gill density/body size | intercept | 1,13 | -6.56 | 1.56 | **0.0010** | 8.3% |
|  | gill density/body size | 1,13 | -0.00359 | 0.00331 | 0.2976 |  |
| PIC: gill density | gill density | 1,13 | -3.149 | 2.936 | 0.3030 | 8.1% |
| PIC: gill density + body size | gill density | 1,12 | -3.081 | 3.034 | 0.3298 | 9.7% |
|  | body size | 1,12 | 1.020 | 2.230 | 0.6555 |  |
| PIC: gill density/body size | gill density/body size | 1,13 | -1.757 | 1.706 | 0.3217 | 7.5% |
| **D) CTmin in 14.5 C acclimated animals** | |  |  |  |  |  |
| OLS: gill density | intercept | 1,13 | -6.28 | 2.82 | **0.0442** | 2.4% |
|  | gill density | 1,13 | -0.00046 | 0.00081 | 0.5821 |  |
| OLS: gill density + body size | intercept | 1,12 | -12.19 | 3.84 | **0.0080** | 27.6% |
|  | gill density | 1,12 | -0.00009 | 0.00075 | 0.9094 |  |
|  | body size | 1,12 | 0.5806 | 0.284 | 0.0633 |  |
| OLS: gill density/body size | intercept | 1,13 | -5.79 | 1.55 | **0.0025** | 12.9% |
|  | gill density/body size | 1,13 | -0.00457 | 0.00329 | 0.1882 |  |
| PIC: gill density | gill density | 1,13 | -0.885 | 3.118 | 0.7810 | 0.6% |
| PIC: gill density + body size | gill density | 1,12 | -0.631 | 2.881 | 0.8303 | 21.9% |
|  | body size | 1,12 | 3.830 | 2.118 | 0.0956 |  |
| PIC: gill density/body size | gill density/body size | 1,13 | -2.687 | 1.651 | 0.1276 | 16.9% |
| **E) delta CTmax** |  |  |  |  |  |  |
| OLS: gill density | intercept | 1,13 | 1.79 | 1.52 | 0.2596 | 8.8% |
|  | gill density | 1,13 | -0.00049 | 0.00044 | 0.2818 |  |
| OLS: gill density + body size | intercept | 1,12 | 1.83 | 2.40 | 0.4617 | 8.8% |
|  | gill density | 1,12 | -0.00049 | 0.00047 | 0.3137 |  |
|  | body size | 1,12 | -0.0035 | 0.178 | 0.9845 |  |
| OLS: gill density/body size | intercept | 1,13 | 0.82 | 0.90 | 0.3812 | 4.9% |
|  | gill density/body size | 1,13 | -0.00158 | 0.00192 | 0.4265 |  |
| PIC: gill density | gill density | 1,13 | -1.795 | 1.854 | 0.3507 | 6.7% |
| PIC: gill density + body size | gill density | 1,12 | -1.824 | 1.924 | 0.3617 | 7.5% |
|  | body size | 1,12 | -0.450 | 1.415 | 0.7561 |  |
| PIC: gill density/body size | gill density/body size | 1,13 | -0.363 | 1.107 | 0.7479 | 0.8% |
| **F) delta CTmin** |  |  |  |  |  |  |
| OLS: gill density | intercept | 1,13 | 0.84 | 1.55 | 0.5983 | 4.3% |
|  | gill density | 1,13 | -0.00034 | 0.00045 | 0.4580 |  |
| OLS: gill density + body size | intercept | 1,12 | 4.49 | 2.02 | **0.0465** | 35.3% |
|  | gill density | 1,12 | -0.00057 | 0.00040 | 0.1737 |  |
|  | body size | 1,12 | -0.3582 | 0.149 | **0.0338** |  |
| OLS: gill density/body size | intercept | 1,13 | -0.77 | 0.92 | 0.4131 | 1.9% |
|  | gill density/body size | 1,13 | 0.00098 | 0.00195 | 0.6219 |  |
| PIC: gill density | gill density | 1,13 | -2.264 | 1.905 | 0.2559 | 9.8% |
| PIC: gill density + body size | gill density | 1,12 | -2.450 | 1.650 | 0.1634 | 37.7% |
|  | body size | 1,12 | -2.810 | 1.213 | **0.0391** |  |
| PIC: gill density/body size | gill density/body size | 1,13 | 0.929 | 1.133 | 0.4267 | 4.9% |
